# Challenges in Medicine: The Odyssey of a Patient with Isolated IgG4-Related Eosinophilic Angiocentric Fibrosis Presenting as a Locally Destructive Sinonasal Mass

**DOI:** 10.1155/2021/6668184

**Published:** 2021-03-09

**Authors:** Snigdha Nutalapati, Richard O'Neal, William O'Connor, Brett T. Comer, Gerhard C. Hildebrandt

**Affiliations:** ^1^University of Kentucky College of Medicine, Division of Hematology and Oncology, Lexington, KY, USA; ^2^University of Kentucky College of Medicine, Department of Pathology and Laboratory Medicine, Lexington, KY, USA; ^3^University of Kentucky College of Medicine, Department of Otolaryngology, Lexington, KY, USA

## Abstract

Eosinophilic angiocentric fibrosis (EAF) is an exceeding rare clinical entity and is considered a part of the spectrum of IgG4-related disease (IgG4RD). We hereby present such an unusual case of a 60-year-old female who presented to us with recurrent sinonasal mass, after a decade long haul of multiple clinical evaluations, biopsies, and debulking surgery without a definitive diagnosis. Over this period, the mass eroded through the ethmoid cells along with nasal septal destruction leading to saddle nose deformity, extended superiorly through the cribriform plates to right frontal lobe, and compressed the optic nerve leading to visual loss. Although initial biopsy was negative, repeat biopsy was performed owing to high clinical suspicion due to all the classic histopathological findings compatible with the diagnosis of eosinophilic angiocentric fibrosis IgG4-related disease (EAF-IgG4RD). Steroids are the recommended first-line therapy; however, our case was resistant to steroids needing rituximab to halt the disease progression. Our case interestingly also had T-cell clonality and isolated isocitrate dehydrogenase 2 enzyme mutation on next-generation sequencing, suggesting a possible role of novel molecular-targeted therapies in this rare disease. This case highlights the clinical challenges physicians face towards diagnosing and treating EAF-IgG4RD, emphasizing the need for high clinical suspicion and the possible role of targeted therapies for this rare disease.

## 1. Introduction

Immunoglobulin G4-related disease (IgG4RD) is a recently recognized systemic disease characterized by immune-mediated fibroinflammation. Although the early descriptions date back to the 1960s, IgG4RD as a unified entity with systemic manifestations was recognized only in 2001 [1, 2]. Eosinophilic angiocentric fibrosis (EAF) is a rare localized tumefactive disease involving upper respiratory tract and orbit, considered to be a part of spectrum of systemic IgG4RD [3]. We hereby present a case of steroid-resistant eosinophilic angiocentric fibrosis IgG4-related disease (EAF-IgG4RD) presenting as an invasive mass-like lesion of ethmoid sinus with T-cell clonality and isocitrate dehydrogenase 2 enzyme (IDH-2) mutation, highlighting the difficulties clinicians face in diagnosing and treating this rare disease.

## 2. Case Presentation

In 2010, a 60-year-old female presented with a right-sided nasopharyngeal mass and imaging revealed soft tissue mass along the inferior nasal cavity with intact ethmoid roof and cribriform plate. Biopsy was nondiagnostic at that time and showed foamy histocytes with acute on chronic inflammatory changes.

Over the following decade, she consulted multiple healthcare providers across the nation as she continued to experience recurrent symptoms of nasal obstruction, epistaxis, hyposmia, nasalization of speech, and sinusitis. Computed tomography (CT) scans revealed progressive local changes with erosion through the ethmoid cells and nasal septal destruction causing saddle nose deformity. She in total underwent 7 biopsies, all reported as nonspecific mixed inflammatory changes with lymphocytic proliferation. Her mass continued to recur, requiring repeated debulking leading to considerable morbidity including eustachian tube dysfunction and right-sided hearing loss.

**Figure 1:**
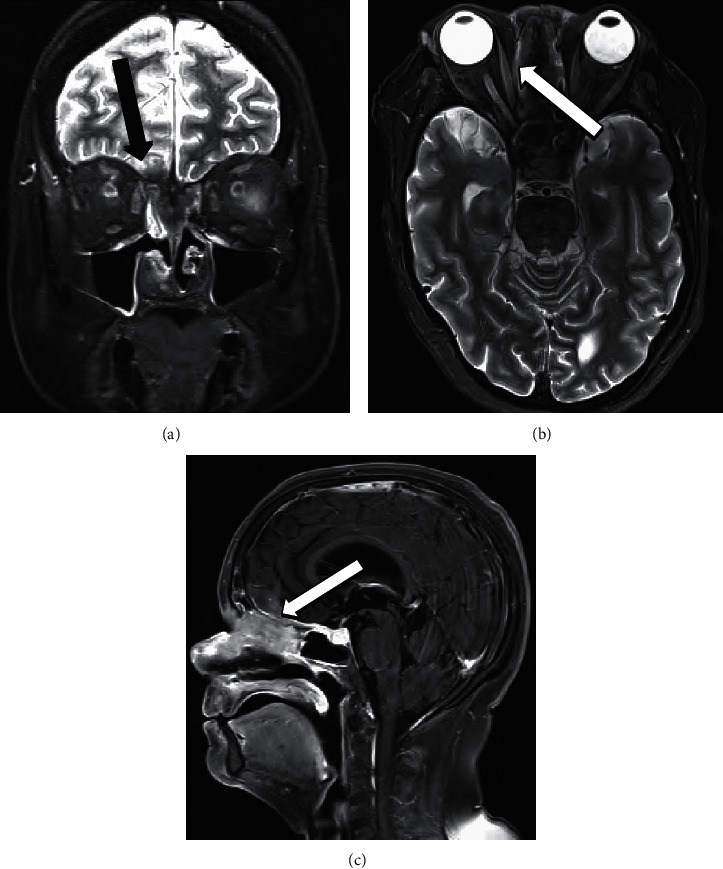
Triplanar MRI coronal STIR (a), axial STIR (b), and sagittal (c) T1-flair with fat saturation postcontrast showing midline anterior skull base mass with extension through skull base and right lamina papyracea (arrows) and saddle nose deformity (star).

**Figure 2:**
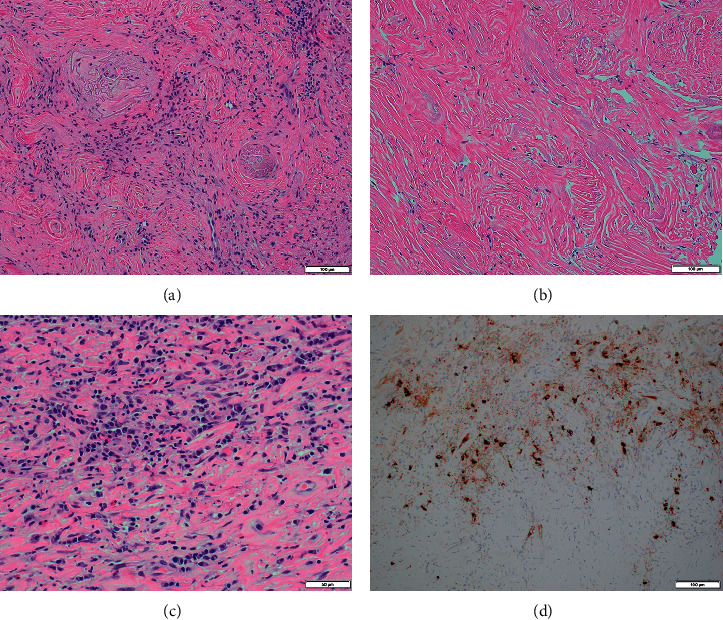
(a) Obliterative phlebitis—20x hematoxylin and eosin (H&E) stain showing cellular fibroinflammatory zone with irregular elastic remnants of larger vein; (b) storiform fibrosis—20x H&E stain showing paucicellular zone of whorled and interlacing collagen bundles with residual spindle-shaped fibroblast nuclei; (c) lymphoplasmacytic infiltration—40x H&E stain showing densely cellular inflammatory infiltrate with numerous plasma cells and eosinophils in a fibrous matrix; (d) immunohistochemistry shows strong positive brown chromogen decoration of IgG4-positive lymphoid cells, exceeding 50/high power field.

She presented to our hospital in late 2019 with complete right eye vision loss and worsening vision in the left eye. Magnetic resonance imaging (MRI) revealed a 4.2 cm mass in the right ethmoid and inferior frontal sinus traversing cribriform plates, compressing bilateral optic nerves, and extending to the right inferior frontal lobe (Figure 1). Serum eosinophil counts were normal at 0.14 k/*μ*L. Serum IgA and IgM were also reported normal 178 mg/dL and 146 mg/dL, respectively. IgG levels were low at 497 mg/dL with normal serum IgG4 levels at 53.9 mg/dL. Repeat biopsy showed mixed acute and chronic inflammatory process with foamy macrophages and background sclerosis. Immunohistochemistry revealed a mixed T-cell and B-cell populations and <10 IgG4-positive plasma cells per high power field (HPF). Serum IgG4 level and peripheral blood flow cytometry were normal. FDG-PET scan showed ethmoid sinus soft tissue mass extending into the right inferior frontal lobe with a SUV of 8.8 and a nodular soft tissue thickening along the left inferior nasal cavity eroding into hard palate with a SUV of 7.5. Due to rapidly deteriorating vision from extrinsic optic nerve compression and edema, she received high-dose IV methylprednisolone 1000 mg for 3 days followed by prednisone 60 mg daily tapered over 8 weeks. Despite transient improvement, her visual loss continued to worsen. In further case discussion, atypical non-Langerhans histiocytosis was considered as a differential diagnosis by pathologists, and she received local radiation to sinonasal mass with 4500 cGy, yet her symptoms did not improve. Of note, next-generation sequencing (NGS) was positive for isolated IDH-2 enzyme mutation and T-cell receptor analysis showed clonality. Repeat FDG-PET postradiation showed no significant change in the size and uptake of the mass. Given unclear etiology and minimal response to steroids and radiation, a repeat biopsy of the mass was pursued. This revealed eosinophilic infiltrates admixed with lymphoplasmacytic inflammatory cells with angiocentric fibrosis, vascular obliteration, and immunohistochemical stains were positive for IgG4 at > 50 cells/HPF, compatible with the diagnosis of IgG4RD (Figure 2). Her disease was deemed steroid refractory, and rituximab was initiated at 1000 mg IV every 15 days for a total of 2 doses. Both her visual symptoms and nasal obstruction began to improve, and sinonasal mass decreased to 2.3 cm on repeat MRI scan.

## 3. Discussion

IgG4RD is immune-mediated systemic fibroinflammatory disease affecting multiple organs, leading to tumefactive tissue destruction [1–4]. EAF is a rare form localized sinonasal IgG4RD, commonly sinonasal cavity and orbits [3]. EAF-IgG4RD has a predilection for local extension associated with osseous destruction and perineural infiltration [3, 5–8]. High clinical suspicion is needed as these are frequently misdiagnosed as inflammatory or cancerous lesions given extensive local invasion and hypermetabolic appearance on FDG-PET scans, delaying appropriate treatment and resulting in unwarranted complications [9, 10].

The three major hallmark histopathological findings of IgG4RD are dense lymphoplasmacytic infiltrate, storiform fibrosis, and obliterative phlebitis. Diagnosis also requires the presence of increased IgG4-positive plasma cells or elevated IgG4 : IgG ratio in the tissue [1]. Although the number of IgG4-positive plasma cells varies based on the organ involved and degree of fibrosis, diffuse infiltration with >50 cells/HPF is considered highly specific for IgG4RD [1, 11]. Proposed to be a form of IgG4RD, histologically EAF-IgG4RD is identified by the distinctive presence of small caliber angiocentric fibrosis and eosinophil-dominant inflammatory infiltrates [3]. Prior biopsies in our case were either reported as nonspecific lacking the hallmark histopathological findings or having <10 IgG4 cells/HPF, not meeting the diagnostic criteria for IgG4RD. At times, sampling noninvolved portion or portion with predominant fibrosis may lead to false-negative results. Also, given the complexity of the diagnosis, histopathological examination should ideally be performed by an experienced pathologist to avoid inconclusive results. As such, if the clinical suspicion is high with no other alternative diagnosis, repeat biopsy should be considered. Final biopsy performed at our institution showed >50 IgG4 cells/HPF along with other required histopathological findings, establishing the diagnosis of EAF-IgG4RD.

T-cells play a significant role in the pathophysiology of IgG4RD including the oligoclonal expansion of CD4 + SLAMF7 + cytotoxic T-lymphocytes (CTLS) and T-follicular helper cells, compatible with the observed T-cell clonality in our patient's tissue biopsy in the absence of morphologic evidence of T-cell lymphoma [12–15]. Of note, heretofore, there is no report on IDH-2 mutation in IgG4RD, which was puzzling for us and, for a moment, raised the concern for a small angioimmunoblastic T-cell lymphoma clone driving plasma cell activation, yet no further supportive evidence was found [16]. The exact meaning of the observed IDH-2 mutation is not clear, yet our case suggests that IDH-2 mutations may be present in IgG4RD and that NGS profiling may help to better understand disease biology and to identify novel molecular therapeutic targets in this rare disease.

Prednisone 0.6 mg/kg/day for a total of 4 weeks followed by taper and discontinuation in 2-3 months is the recommended treatment for IgG4RD [17]. This is largely based on expert opinions and from clinical experiences in treating autoimmune pancreatitis. Rituximab was shown to be a promising alternative agent with an overall response rate of 83% even among cases resistant to steroids and immunomodulators [18–22]. Currently recommended dose regimen is 1000 mg IV every 15 days for a total of 2 doses. Given aggressive local destruction, intracranial invasion with vision loss, and recrudescence with initial steroid therapy, we opted for rituximab as a steroid alternative treatment option in our case and achieved desired clinical response. Use of rituximab in combination with fludarabine or bendamustine was also reported especially among cases of IgG4RD coexisting hyperviscosity syndrome [3, 23–25]. Use of rituximab alone achieved desired clinical response in our case without the need for alternative regimens.

EAF-IgG4RD can be challenging to diagnose, and a high index of clinical suspicion followed by focused clinicopathological examination is the key for establishing the correct diagnosis. Our case reflects a 10-year journey similar to Odysseus' return to Ithaca after the Trojan War. Whether in addition to current imaging and histopathology, the detection of molecular targets such as the observed IDH-2 mutation will be helpful in diagnosis, and management of IgG4RD warrants to be explored further, as subtle coincidental findings often mean a lot, similar to Eurycleia recognizing Odysseus by his scar above the knee (Odyssey 19.386-507).

## Data Availability

No data were used to support this study.

## References

[1] Deshpande V., Zen Y., Chan J. K. (2012). Consensus statement on the pathology of IgG4-related disease. *Modern Pathology: An Official Journal of the United States and Canadian Academy of Pathology, Inc*.

[2] Maehara T., Moriyama M., Nakamura S. (2020). Review of a novel disease entity, immunoglobulin G4-related disease. *Journal of the Korean Association of Oral and Maxillofacial Surgeons*.

[3] Deshpande V., Khosroshahi A., Nielsen G. P., Hamilos D. L., Stone J. H. (2011). Eosinophilic angiocentric fibrosis is a form of IgG4-related systemic disease. *American Journal of Surgical Pathology*.

[4] Vandjelovic N. D., Humphreys I. M. (2016). Immunoglobulin G4-related sclerosing disease of the paranasal sinuses: a case report and literature review. *Allergy & Rhinology*.

[5] Ahn J., Flanagan M. (2018). Eosinophilic angiocentric fibrosis: a review and update of its association with immunoglobulin G4-related disease. *Archives of Pathology & Laboratory Medicine*.

[6] Yang B. T., Wang Y. Z., Wang X. Y., Wang Z. C. (2011). Nasal cavity eosinophilic angiocentric fibrosis: CT and MR imaging findings. *American Journal of Neuroradiology*.

[7] Sunde J., Alexander K. A., Reddy V. V. B., Woodworth B. A. (2010). Intranasal eosinophilic angiocentric fibrosis: a case report and review. *Head and Neck Pathology*.

[8] Narayan J., Douglas-Jones A. G. (2005). Eosinophilic angiocentric fibrosis and granuloma faciale: analysis of cellular infiltrate and review of literature. *Annals of Otology, Rhinology & Laryngology*.

[9] Pace C., Ward S. (2010). A rare case of IgG4-related sclerosing disease of the maxillary sinus associated with bone destruction. *Journal of Oral and Maxillofacial Surgery*.

[10] Inoue A., Wada K., Matsuura K. (2016). IgG4-related disease in the sinonasal cavity accompanied by intranasal structure loss. *Auris Nasus Larynx*.

[11] Zen Y., Nakanuma Y. (2010). IgG4-Related disease. *American Journal of Surgical Pathology*.

[12] Mattoo H., Mahajan V. S., Maehara T. (2016). Clonal expansion of CD4 + cytotoxic T lymphocytes in patients with IgG 4 -related disease. *Journal of Allergy and Clinical Immunology*.

[13] Akiyama M., Suzuki K., Yasuoka H., Kaneko Y., Yamaoka K., Takeuchi T. (2018). Follicular helper T cells in the pathogenesis of IgG4-related disease. *Rheumatology*.

[14] Mattoo H., Stone J. H., Pillai S. (2017). Clonally expanded cytotoxic CD4+ T cells and the pathogenesis of IgG4-related disease. *Autoimmunity*.

[15] Wang L., Zhang P., Li J. (2019). High-throughput sequencing of CD4+ T cell repertoire reveals disease-specific signatures in IgG4-related disease. *Arthritis Research & Therapy*.

[16] Cairns R. A., Iqbal J., Lemonnier F. (2012). IDH2 mutations are frequent in angioimmunoblastic T-cell lymphoma. *Blood*.

[17] Khosroshahi A., Wallace Z. S., Crowe J. L. (2015). International consensus guidance statement on the management and treatment of IgG4-related disease. *Arthritis & Rheumatology*.

[18] Carruthers M. N., Topazian M. D., Khosroshahi A. (2015). Rituximab for IgG4-related disease: a prospective, open-label trial. *Annals of the Rheumatic Diseases*.

[19] Hart P. A., Topazian M. D., Witzig T. E. (2013). Treatment of relapsing autoimmune pancreatitis with immunomodulators and rituximab: the Mayo Clinic experience. *Gut*.

[20] Khosroshahi A., Bloch D. B., Deshpande V., Stone J. H. (2010). Rituximab therapy leads to rapid decline of serum IgG4 levels and prompt clinical improvement in IgG4-related systemic disease. *Arthritis & Rheumatism*.

[21] Khosroshahi A., Stone J. H. (2011). Treatment approaches to IgG4-related systemic disease. *Current Opinion in Rheumatology*.

[22] Khosroshahi A., Carruthers M. N., Deshpande V., Unizony S., Bloch D. B., Stone J. H. (2012). Rituximab for the treatment of IgG4-related disease. *Medicine*.

[23] Chen L. Y. C., Wong P. C. W., Noda S., Collins D. R., Sreenivasan G. M., Coupland R. C. (2015). Polyclonal hyperviscosity syndrome in IgG4‐related disease and associated conditions. *Clinical Case Reports*.

[24] Karmali R. (2020). A challenging case of IgG4-related dis-ease with multiple relapses: effective treatment with bendamustine and rituximab. *Clinics of Oncology*.

[25] Wong P. C. W., Fung A. T., Gerrie A. S. (2013). IgG4-related disease with hypergammaglobulinemic hyperviscosity and retinopathy. *European Journal of Haematology*.

